# Supplementary material for: Impacts of cellulase and xylanase addition on antibiotic resistance and microbial community during dairy manure composting
Source: PLoS One. 2025 Aug 29;20(8):e0328055. doi: 10.1371/journal.pone.0328055 (PMC12396641; doi:10.1371/journal.pone.0328055)
Supplement: S1 File — (DOCX) [file pone.0328055.s001.docx]

**S1 File.**

**Primer list used in high-throughput quantitative PCR (HT-qPCR).** A total of 296 primer pairs including 285 pairs targeting antibiotic resistance genes, 10 pairs targeting mobile genetic elements (MGEs), and 1 pair targeting the 16S rDNA gene.

No. Gene Name Forward Primer Reverse Primer

1. 16S rRNA GGGTTGCGCTCGTTGC ATGGYTGTCGTCAGCTCGTG
2. aac CCCTGCGTTGTGGCTATGT TTGGCCACGCCAATCC
3. aac(6')I1 GACCGGATTAAGGCCGATG CTTGCCTTGATATTCAGTTTTTATAACCA
4. aac(6')-Ib(aka aacA4)-01 GTTTGAGAGGCAAGGTACCGTAA GAATGCCTGGCGTGTTTGA
5. aac(6')-Ib(aka aacA4)-02 CGTCGCCGAGCAACTTG CGGTACCTTGCCTCTCAAACC
6. aac(6')-Ib(aka aacA4)-03 AGAAGCACGCCCGACACTT GCTCTCCATTCAGCATTGCA
7. aac(6')-II CGACCCGACTCCGAACAA GCACGAATCCTGCCTTCTCA
8. aac(6')-Iy GCTTTGCGGATGCCTCAAT GGAGAACAAAAATACCTTCAAGGAAA
9. aacA/aphD AGAGCCTTGGGAAGATGAAGTTT TTGATCCATACCATAGACTATCTCATCA
10. aacC CGTCACTTATTCGATGCCCTTAC GTCGGGCGCGGCATA
11. aacC1 GGTCGTGAGTTCGGAGACGTA GCAAGTTCCCGAGGTAATCG
12. aacC2 ACGGCATTCTCGATTGCTTT CCGAGCTTCACGTAAGCATTT
13. aacC4 CGGCGTGGGACACGAT AGGGAACCTTTGCCATCAACT
14. aadA-01 GTTGTGCACGACGACATCATT GGCTCGAAGATACCTGCAAGAA
15. aadA-02 CGAGATTCTCCGCGCTGTA GCTGCCATTCTCCAAATTGC
16. aadA1 AGCTAAGCGCGAACTGCAAT TGGCTCGAAGATACCTGCAA
17. aadA-1-01 AAAAGCCCGAAGAGGAACTTG CATCTTTCACAAAGATGTTGCTGTCT
18. aadA-1-02 CGGAATTGAAAAAACTGATCGAA ATACCGGCTGTCCGTCATTT
19. aadA2-01 ACGGCTCCGCAGTGGAT GGCCACAGTAACCAACAAATCA
20. aadA2-02 CTTGTCGTGCATGACGACATC TCGAAGATACCCGCAAGAATG
21. aadA2-03 CAATGACATTCTTGCGGGTATC GACCTACCAAGGCAACGCTATG
22. aadA5-01 ATCACGATCTTGCGATTTTGCT CTGCGGATGGGCCTAGAAG
23. aadA5-02 GTTCTTGCTCTTGCTCGCATT GATGCTCGGCAGGCAAAC
24. aadA9-01 CGCGGCAAGCCTATCTTG CAAATCAGCGACCGCAGACT
25. aadA9-02 GGATGCACGCTTGGATGAA CCTCTAGCGGCCGGAGTATT
26. aadD CCGACAACATTTCTACCATCCTT ACCGAAGCGCTCGTCGTATA
27. aadE TACCTTATTGCCCTTGGAAGAGTTA GGAACTATGTCCCTTTTAATTCTACAATCT
28. acrA-01 CAACGATCGGACGGGTTTC TGGCGATGCCACCGTACT
29. acrA-02 GGTCTATCACCCTACGCGCTATC GCGCGCACGAACATACC
30. acrA-03 CAGACCCGCATCGCATATT CGACAATTTCGCGCTCATG
31. acrA-04 TACTTTGCGCGCCATCTTC CGTGCGCGAACGAACAT
32. acrA-05 CGTGCGCGAACGAACA ACTTTGCGCGCCATCTTC
33. acrB-01 AGTCGGTGTTCGCCGTTAAC CAAGGAAACGAACGCAATACC
34. acrF GCGGCCAGGCACAAAA TACGCTCTTCCCACGGTTTC
35. acrR-01 GCGCTGGAGACACGACAAC GCCTTGCTGCGAGAACAAA
36. acrR-02 GATGATACCCCCTGCTGTGAGA ACCAAACAAGAAGCGCAAGAA
37. adeA CAGTTCGAGCGCCTATTTCTG CGCCCTGACCGACCAAT
38. ampC/blaDHA TGGCCGCAGCAGAAAGA CCGTTTTATGCACCCAGGAA
39. ampC-01 TGGCGTATCGGGTCAATGT CTCCACGGGCCAGTTGAG
40. ampC-02 GCAGCACGCCCCGTAA TGTACCCATGATGCGCGTACT
41. ampC-04 TCCGGTGACGCGACAGA CAGCACGCCGGTGAAAGT
42. ampC-05 CTGTTCGAGCTGGGTTCTATAAGTAAA CAGTATCTGGTCACCGGATCGT
43. ampC-06 CCGCTCAAGCTGGACCATAC CCATATCCTGCACGTTGGTTT
44. ampC-07 CCGCCCAGAGCAAGGACTA GCTCGACTTCACGCCGTAAG
45. ampC-09 CAGCCGCTGATGAAAAAATATG CAGCGAGCCCACTTCGA
46. aph TTTCAGCAAGTGGATCATGTTAAAAT CCAAGCTGTTTCCACTGTTTTTC
47. aph(2')-Id-01 TGAGCAGTATCATAAGTTGAGTGAAAAG GACAGAACAATCAATCTCTATGGAATG
48. aph(2')-Id-02 TAAGGATATACCGACAGTTTTGGAAA TTTAATCCCTCTTCATACCAATCCATA
49. aph6ia CCCATCCCATGTGTAAGGAAA GCCACCGCTTCTGCTGTAC
50. aphA1(aka kanR) TGAACAAGTCTGGAAAGAAATGCA CCTATTAATTTCCCCTCGTCAAAAA
51. bacA-01 CGGCTTCGTGACCTCGTT ACAATGCGATACCAGGCAAAT
52. bacA-02 TTCCACGACACGATTAAGTCATTG CGGCTCTTTCGGCTTCAG
53. bla1 GCAAGTTGAAGCGAAAGAAAAGA TACCAGTATCAATCGCATATACACCTAA
54. bla-ACC-1 CACACAGCTGATGGCTTATCTAAAA AATAAACGCGATGGGTTCCA
55. blaCMY CCGCGGCGAAATTAAGC GCCACTGTTTGCCTGTCAGTT
56. blaCMY2-01 AAAGCCTCAT GGGTGCATAAA ATAGCTTTTGTTTGCCAGCATCA
57. blaCMY2-02 GCGAGCAGCCTGAAGCA CGGATGGGCTTGTCCTCTT
58. blaCTX-M-01 GGAGGCGTGACGGCTTTT TTCAGTGCGATCCAGACGAA
59. blaCTX-M-02 GCCGCGGTGCTGAAGA ATCGGATTATAGTTAACCAGGTCAGATTT
60. blaCTX-M-03 CGATACCACCACGCCGTTA GCATTGCCCAACGTCAGATT
61. blaCTX-M-04 CTTGGCGTTGCGCTGAT CGTTCATCGGCACGGTAGA
62. blaCTX-M-05 GCGATAACGTGGCGATGAAT GTCGAGACGGAACGTTTCGT
63. blaCTX-M-06 CACAGTTGGTGACGTGGCTTAA CTCCGCTGCCGGTTTTATC
64. blaGES GCAATGTGCTCAACGTTCAAG GTGCCTGAGTCAATTCTTTCAAAG
65. blaIMP-01 AACACGGTTTGGTGGTTCTTGTA GCGCTCCACAAACCAATTG
66. blaIMP-02 AAGGCAGCATTTCCTCTCATTTT GGATAGATCGAGAATTAAGCCACTCT
67. bla-L1 CACCGGGTTACCAGCTGAAG GCGAAGCTGCGCTTGTAGTC
68. blaMOX/blaCMY CTATGTCAATGTGCCGAAGCA GGCTTGTCCTCTTTCGAATAGC
69. blaOCH GGCGACTTGCGCCGTAT TTTTCTGCTCGGCCATGAG
70. blaOKP GCCGCCATCACCATGAG GGTGACGTTGTCACCGATCTG
71. blaOXA1/blaOXA30 CGGATGGTTTGAAGGGTTTATTAT TCTTGGCTTTTATGCTTGATGTTAA
72. blaOXA10-01 CGCAATTATCGGCCTAGAAACT TTGGCTTTCCGTCCCATTT
73. blaOXA10-02 CGCAATTATCGGCCTAGAAACT TTGGCTTTCCGTCCCATTT
74. blaOXY CGTTCAGGCGGCAGGTT GCCGCGATATAAGATTTGAGAATT
75. blaPAO CGCCGTACAACCGGTGAT GAAGTAATGCGGTTCTCCTTTCA
76. blaPER TGCTGGTTGCTGTTTTTGTGA CCTGCGCAATGATAGCTTCAT
77. blaPSE TTGTGACCTATTCCCCTGTAATAGAA TGCGAAGCACGCATCATC
78. blaROB GCAAAGGCATGACGATTGC CGCGCTGTTGTCGCTAAA
79. blaSFO CCGCCGCCATCCAGTA GGGCCGCCAAGATGCT
80. blaSHV-01 TCCCATGATGAGCACCTTTAAA TTCGTCACCGGCATCCA
81. blaSHV-02 CTTTCCCATGATGAGCACCTTT TCCTGCTGGCGATAGTGGAT
82. blaTEM AGCATCTTACGGATGGCATGA TCCTCCGATCGTTGTCAGAAGT
83. blaTLA ACACTTTGCCATTGCTGTTTATGT TGCAAATTTCGGCAATAATCTTT
84. blaVEB CCCGATGCAAAGCGTTATG GAAAGATTCCCTTTATCTATCTCAGACAA
85. blaVIM GCACTTCTCGCGGAGATTG CGACGGTGATGCGTACGTT
86. blaZ GGAGATAAAGTAACAAATCCAGTTAGATATGA TGCTTAATTTTCCATTTGCGATAAG
87. carB GGAGTGAGGCTGACCGTAGAAG ATCGGCGAAACGCACAAA
88. catA1 GGGTGAGTTTCACCAGTTTTGATT CACCTTGTCGCCTTGCGTATA
89. catB3 GCACTCGATGCCTTCCAAAA AGAGCCGATCCAAACGTCAT
90. catB8 CACTCGACGCCTTCCAAAG CCGAGCCTATCCAGACATCATT
91. ceoA ATCAACACGGACCAGGACAAG GGAAAGTCCGCTCACGATGA
92. cepA AGTTGCGCAGAACAGTCCTCTT TCGTATCTTGCCCGTCGATAAT
93. cfiA GCAGCGTTGCTGGACACA GTTCGGGATAAACGTGGTGACT
94. cfr GCAAAATTCAGAGCAAGTTACGAA AAAATGACTCCCAACCTGCTTTAT
95. cfxA TCATTCCTCGTTCAAGTTTTCAGA TGCAGCACCAAGAGGAGATGT
96. cIntI-1(class1) GGCATCCAAGCAGCAAG AAGCAGACTTGACCTGA
97. cmeA GCAGCAAAGAAGAAGCACCAA AGCAGGGTAAGTAAAACTAAGTGGTAAATCT
98. cmlA1-01 TAGGAAGCATCGGAACGTTGAT CAGACCGAGCACGACTGTTG
99. cmlA1-02 AGGAAGCATCGGAACGTTGA ACAGACCGAGCACGACTGTTG
100. cmr CGGCATCGTCAGTGGAATT CGGTTCCGAAAAAGATGGAA
101. cmx(A) GCGATCGCCATCCTCTGT TCGACACGGAGCCTTGGT
102. cphA-01 GCGAGCTGCACAAGCTGAT CGGCCCAGTCGCTCTTC
103. cphA-02 GTGCTGATGGCGAGTTTCTG GGTGTGGTAGTTGGTGTTGATCAC
104. dfrA1 GGAATGGCCCTGATATTCCA AGTCTTGCGTCCAACCAACAG
105. dfrA12 CCTCTACCGAACCGTCACACA GCGACAGCGTTGAAACAACTAC
106. emrD CTCAGCAGTATGGTGGTAAGCATT ACCAGGCGCCGAAGAAC
107. ereA CCTGTGGTACGGAGAATTCATGT ACCGCATTCGCTTTGCTT
108. ereB GCTTTATTTCAGGAGGCGGAAT TTTTAAATGCCACAGCACAGAATC
109. erm(34) GCGCGTTGACGACGATTT TGGTCATACTCGACGGCTAGAAC
110. erm(35) TTGAAAACGATGTTGCATTAAGTCA TCTATAATCACAACTAACCACTTGAACGT
111. erm(36) GGCGGACCGACTTGCAT TCTGCGTTGACGACGGTTAC
112. ermA TTGAGAAGGGATTTGCGAAAAG ATATCCATCTCCACCATTAATAGTAAACC
113. ermA/ermTR ACATTTTACCAAGGAACTTGTGGAA GTGGCATGACATAAACCTTCATCA
114. ermB TAAAGGGCATTTAACGACGAAACT TTTATACCTCTGTTTGTTAGGGAATTGAA
115. ermC TTTGAAATCGGCTCAGGAAAA ATGGTCTATTTCAATGGCAGTTACG
116. ermF CAGCTTTGGTTGAACATTTACGAA AAATTCCTAAAATCACAACCGACAA
117. ermJ/ermD GGACTCGGCAATGGTCAGAA CCCCGAAACGCAATATAATGTT
118. ermK-01 GTTTGATATTGGCATTGTCAGAGAAA ACCATTGCCGAGTCCACTTT
119. ermK-02 GAGCCGCAAGCCCCTTT GTGTTTCATTTGACGCGGAGTAA
120. ermT-01 GTTCACTAGCACTATTTTTAATGACAGAAGT GAAGGGTGTCTTTTTAATACAATTAACGA
121. ermT-02 GTAAAATCCCTAGAGAATACTTTCATCCA TGAGTGATATTTTTGAAGGGTGTCTT
122. ermX GCTCAGTGGTCCCCATGGT ATCCCCCCGTCAACGTTT
123. ermY TTGTCTTTGAAAGTGAAGCAACAGT TAACGCTAGAGAACGATTTGTATTGAG
124. fabK TTTCAGCTCAGCACTTTGGTCAT AAGGCATCTTTTTCAGCCAGTTC
125. floR ATTGTCTTCACGGTGTCCGTTA CCGCGATGTCGTCGAACT
126. folA CGAGCAGTTCCTGCCAAAG CCCAGTCATCCGGTTCATAATC
127. fosB TCACTGTAACTAATGAAGCATTAGACCAT CCATCTGGATCTGTAAAGTAAAGAGATC
128. fos X GATTAAGCCATATCACTTTAATTGTGAAAG TCTCCTTCCATAATGCAAATCCA
129. fox5 GGTTTGCCGCTGCAGTTC GCGGCCAGGTGACCAA
130. imiR CCGGACTAGAGCTTCATGTAAGC CCCACGCGGTACTCTTGTAAA
131. intI-1(clinic) CGAACGAGTGGCGGAGGGTG TACCCGAGAGCTTGGCACCCA
132. IS613 AGGTTCGGACTCAATGCAACA TTCAGCACATACCGCCTTGAT
133. lmrA-01 TCGACGTGACCGTAGTGAACA CGTGACTACCCAGGTGAGTTGA
134. lnuA-01 TGACGCTCAACACACTCAAAAA TTCATGCTTAAGTTCCATACGTGAA
135. lnuB-01 TGAACATAATCCCCTCGTTTAAAGAT TAATTGCCCTGTTTCATCGTAAATAA
136. lnuB-02 AAAGGAGAAGGTGACCAATACTCTGA GGAGCTACGTCAAACAACCAGTT
137. lnuC TGGTCAATATAACAGATGTAAACCAGATTT CACCCCAGCCACCATCAA
138. marR-01 GCGGCGTACTGGTGAAGCTA TGCCCTGGTCGTTGATGA
139. matA/mel TAGTAGGCAAGCTCGGTGTTGA CCTGTGCTATTTTAAGCCTTGTTTCT
140. mdetl1 ATACAGCAGTGGATATTGGTTTAATTGT TGCATAAGGTGAATGTTCCATGA
141. mdtA CCTAACGGGCGTGACTTCA TTCACCTGTTTCAAGGGTCAAA
142. mdtE/yhiU CGTCGGCGCACTCGTT TCCAGACGTTGTACGGTAACCA
143. mecA GGTTACGGACAAGGTGAAATACTGAT TGTCTTTTAATAAGTGAGGTGCGTTAATA
144. mefA CCGTAGCATTGGAACAGCTTTT AAACGGAGTATAAGAGTGCTGCAA
145. mepA ATCGGTCGCTCTTCGTTCAC ATAAATAGGATCGAGCTGCTGGAT
146. mexA AGGACAACGCTATGCAACGAA CCGGAAAGGGCCGAAAT
147. mexD TTGCCACTGGCTTTCATGAG CACTGCGGAGAACTGTCTGTAGA
148. mexE GGTCAGCACCGACAAGGTCTAC AGCTCGACGTACTTGAGGAACAC
149. mexF CCGCGAGAAGGCCAAGA TTGAGTTCGGCGGTGATGA
150. mphA-01 CTGACGCGCTCCGTGTT GGTGGTGCATGGCGATCT
151. mphA-02 TGATGACCCTGCCATCGA TTCGCGAGCCCCTCTTC
152. mphB CGCAGCGCTTGATCTTGTAG TTACTGCATCCATACGCTGCTT
153. mphC CGTTTGAAGTACCGAATTGGAAA GCTGCGGGTTTGCCTGTA
154. msrA-01 CTGCTAACACAAGTACGATTCCAAAT TCAAGTAAAGTTGTCTTACCTACACCATT
155. msrC-01 TCAGACCGGATCGGTTGTC CCTATTTTTTGGAGTCTTCTCTCTAATGTT
156. mtrC-01 GGACGGGAAGATGGTCCAA CGTAGCGTTCCGGTTCGAT
157. mtrC-02 CGGAGTCCATCGACCATTTG ATCGTCGGCAAGGAGAATCA
158. mtrD-02 GGTCGGCACGCTCTTGTC TGAAGAATTTGCGCACCACTAC
159. mtrD-03 CCGCCAAGCCGATATAGACA GGCCGGGTTGCCAAA
160. ndm-1 ATTAGCCGCTGCATTGAT CATGTCGAGATAGGAAGTG
161. nimE TGCGCCAAGATAGGGCATA GTCGTGAATTCGGCAGGTTTA
162. nisB GGGAGAGTTGCCGATGTTGTA AGCCACTCGTTAAAGGGCAAT
163. oleC CCCGGAGTCGATGTTCGA GCCGAAGACGTACACGAACAG
164. oprD ATGAAGTGGAGCGCCATTG GGCCACGGCGAACTGA
165. oprJ ACGAGAGTGGCGTCGACAA AAGGCGATCTCGTTGAGGAA
166. pbp CCGGTGCCATTGGTTTAGA AAAATAGCCGCCCCAAGATT
167. pbp2x TTTCATAAGTATCTGGACATGGAAGAA CCAAAGGAAACTTGCTTGAGATTAG
168. Pbp5 GGCGAACTTCTAATTAATCCTATCCA CGCCGATGACATTCTTCTTATCTT
169. penA AGACGGTAACGTATAACTTTTTGAAAGA GCGTGTAGCCGGCAATG
170. pikR1 TCGACATGCGTGACGAGATT CCGCGAATTAGGCCAGAA
171. pikR2 TCGTGGGCCAGGTGAAGA TTCCCCTTGCCGGTGAA
172. pmrA TTTGCAGGTTTTGTTCCTAATGC GCAGAGCCTGATTTCTCCTTTG
173. pncA GCAATCGAGGCGGTGTTC TTGCCGCAGCCAATTCA
174. putitive multidrug AATTTTGCCGATTATTGCTGAAA GATTGTCATCATTCGTTTATCACCAA
175. qac CAATAATAACCGAAATAATAGGGACAAGTT AATAAGTGTTCCTAGTGTTGGCCATAG
176. qacA TGGCAATAGGAGCTATGGTGTTT AAGGTAACACTATTTTCGGTCCAAATC
177. qacA/qacB TTTAGGCAGCCTCGCTTCA CCGAATCCAAATAAAACCCAATAA
178. qacEdelta1-01 TCGCAACATCCGCATTAAAA ATGGATTTCAGAACCAGAGAAAGAAA
179. qacEdelta1-02 CCCCTTCCGCCGTTGT CGACCAGACTGCATAAGCAACA
180. qacH-01 GTGGCAGCTATCGCTTGGAT CCAACGAACGCCCACAA
181. qacH-02 CATCGTGCTTGTGGCAGCTA TGAACGCCCAGAAGTCTAGTTTT
182. qnrA AGGATTTCTCACGCCAGGATT CCGCTTTCAATGAAACTGCAA
183. rarD-02 TGACGCATCGCGTGATCT AAATTTTCTGTGGCGTCTGAATC
184. sat4 GAATGGGCAAAGCATAAAAACTTG CCGATTTTGAAACCACAATTATGATA
185. sdeB CACTACCGCTTCCGCACTTAA TGAAAAAACGGGAAAAGTCCAT
186. spcN-01 AAAAGTTCGATGAAACACGCCTAT TCCAGTGGTAGTCCCCGAATC
187. spcN-02 CAGAATCTTCCTGAAAAGTTTGATGAA CGCAGACACGCCGAATC
188. speA GCAAGAGGTATTTGCTCAACAAGA CAGGGTCACCCTCATAAAGAAAA
189. str AATGAGTTTTGGAGTGTCTCAACGTA AATCAAAACCCCTATTAAAGCCAAT
190. strA CCGGTGGCATTTGAGAAAAA GTGGCTCAACCTGCGAAAAG
191. strB GCTCGGTCGTGAGAACAATCT CAATTTCGGTCGCCTGGTAGT
192. sul1 CAGCGCTATGCGCTCAAG ATCCCGCTGCGCTGAGT
193. sul2 TCATCTGCCAAACTCGTCGTTA GTCAAAGAACGCCGCAATGT
194. sulA/folP-01 CAGGCTCGTAAATTGATAGCAGAAG CTTTCCTTGCGAATCGCTTT
195. sulA/folP-03 CACGGCTTCGGCTCATGT TGCCATCCTGTGACTAGCTACGT
196. tet(32) CCATTACTTCGGACAACGGTAGA CAATCTCTGTGAGGGCATTTAACA
197. tet(34) CTTAGCGCAAACAGCAATCAGT CGGTGATACAGCGCGTAAACT
198. tet(35) ACCCCATGACGTACCTGTAGAGA CAACCCACACTGGCTACCAGTT
199. tet(36)-01 AGAATACTCAGCAGAGGTCAGTTCCT TGGTAGGTCGATAACCCGAAAAT
200. tet(36)-02 TGCAGGAAAGACCTCCATTACAG CTTTGTCCACACTTCCACGTACTATG
201. tet(37) GAGAACGTTGAAAAGGTGGTGAA AACCAAGCCTGGATCAGTCTCA
202. tetA-01 GCTGTTTGTTCTGCCGGAAA GGTTAAGTTCCTTGAACGCAAACT
203. tetA-02 CTCACCAGCCTGACCTCGAT CACGTTGTTATAGAAGCCGCATAG
204. tetB-01 AGTGCGCTTTGGATGCTGTA AGCCCCAGTAGCTCCTGTGA
205. tetB-02 GCCCAGTGCTGTTGTTGTCAT TGAAAGCAAACGGCCTAAATACA
206. tetC-01 CATATCGCAATACATGCGAAAAA AAAGCCGCGGTAAATAGCAA
207. tetC-02 ACTGGTAAGGTAAACGCCATTGTC ATGCATAAACCAGCCATTGAGTAAG
208. tetD-01 TGCCGCGTTTGATTACACA CACCAGTGATCCCGGAGATAA
209. tetD-02 TGTCATCGCGCTGGTGATT CATCCGCTTCCGGGAGAT
210. tetE TTGGCGCTGTATGCAATGAT CGACGACCTATGCGATCTGA
211. tetG-01 TCAACCATTGCCGATTCGA TGGCCCGGCAATCATG
212. tetG-02 CATCAGCGCCGGTCTTATG CCCCATGTAGCCGAACCA
213. tetH TTTGGGTCATCTTACCAGCATTAA TTGCGCATTATCATCGACAGA
214. tetJ GGGTGCCGCATTAGATTACCT TCGTCCAATGTAGAGCATCCATA
215. tetK CAGCAGTCATTGGAAAATTATCTGATTATA CCTTGTACTAACCTACCAAAAATCAAAATA
216. tetL-01 AGCCCGATTTATTCAAGGAATTG CAAATGCTTTCCCCCTGTTCT
217. tetL-02 ATGGTTGTAGTTGCGCGCTATAT ATCGCTGGACCGACTCCTT
218. tetM-01 CATCATAGACACGCCAGGACATAT CGCCATCTTTTGCAGAAATCA
219. tetM-02 TAATATTGGAGTTTTAGCTCATGTTGATG CCTCTCTGACGTTCTAAAAGCGTATTAT
220. tetO-01 ATGTGGATACTACAACGCATGAGATT TGCCTCCACATGATATTTTTCCT
221. tetPA AGTTGCAGATGTGTATAGTCGTAAACTATCTATT TGCTACAAGTACGAAAACAAAACTAGAA
222. tetPB-01 ACACCTGGACACGCTGATTTT ACCGTCTAGAACGCGGAATG
223. tetPB-02 TGATACACCTGGACACGCTGAT CGTCCAAAACGCGGAATG
224. tetPB-03 TGGGCGACAGTAGGCTTAGAA TGACCCTACTGAAACATTAGAAATATACCT
225. tetPB-04 AGTGGTGCAAATACTGAAAAAGTTGT TTTGTTCCTTCGTTTTGGACAGA
226. tetPB-05 CTGAAGTGGAGCGATCATTCC CCCTCAACGGCAGAAATAACTAA
227. tetQ CGCCTCAGAAGTAAGTTCATACACTAAG TCGTTCATGCGGATATTATCAGAAT
228. tetR-02 CGCGATAGACGCCTTCGA TCCTGACAACGAGCCTCCTT
229. tetR-03 CGCGATGGAGCAAAAGTACAT AGTGAAAAACCTTGTTGGCATAAAA
230. tetS TTAAGGACAAACTTTCTGACGACATC TGTCTCCCATTGTTCTGGTTCA
231. tetT CCATATAGAGGTTCCACCAAATCC TGACCCTATTGGTAGTGGTTCTATTG
232. tetU-01 GTGGCAAAGCAACGGATTG TGCGGGCTTGCAAAACTATC
233. tetV GCGGGAACGACGATGTATATC CCGCTATCTCACGACCATGAT
234. tetX AAATTTGTTACCGACACGGAAGTT CATAGCTGAAAAAATCCAGGACAGTT
235. tnpA-01 CATCATCGGACGGACAGAATT GTCGGAGATGTGGGTGTAGAAAGT
236. tnpA-02 GGGCGGGTCGATTGAAA GTGGGCGGGATCTGCTT
237. tnpA-03 AATTGATGCGGACGGCTTAA TCACCAAACTGTTTATGGAGTCGTT
238. tnpA-04 CCGATCACGGAAAGCTCAAG GGCTCGCATGACTTCGAATC
239. tnpA-05 GCCGCACTGTCGATTTTTATC GCGGGATCTGCCACTTCTT
240. tnpA-07 GAAACCGATGCTACAATATCCAATTT CAGCACCGTTTGCAGTGTAAG
241. tolC-01 GGCCGAGAACCTGATGCA AGACTTACGCAATTCCGGGTTA
242. tolC-02 CAGGCAGAGAACCTGATGCA CGCAATTCCGGGTTGCT
243. tolC-03 GCCAGGCAGAGAACCTGATG CGCAATTCCGGGTTGCT
244. Tp614 GGAAATCAACGGCATCCAGTT CATCCATGCGCTTTTGTCTCT
245. ttgA ACGCCAATGCCAAACGATT GTCACGGCGCAGCTTGA
246. ttgB TCGCCCTGGATGTACACCTT ACCATTGCCGACATCAACAAC
247. vanA AAAAGGCTCTGAAAACGCAGTTAT CGGCCGTTATCTTGTAAAAACAT
248. vanB-01 TTGTCGGCGAAGTGGATCA AGCCTTTTTCCGGCTCGTT
249. vanB-02 CCGGTCGAGGAACGAAATC TCCTCCTGCAAAAAAAGATCAAC
250. vanC-01 ACAGGGATTGGCTATGAACCAT TGACTGGCGATGATTTGACTATG
251. vanC-03 AAATCAATACTATGCCGGGCTTT CCGACCGCTGCCATCA
252. vanC1 AGGCGATAGCGGGTATTGAA CAATCGTCAATTGCTCATTTCC
253. vanC2/vanC3 TTTGACTGTCGGTGCTTGTGA TCAATCGTTTCAGGCAATGG
254. vanG ATTTGAATTGGCAGGTATACAGGTTA TGATTTGTCTTTGTCCATACATAATGC
255. vanHB GAGGTTTCCGAGGCGACAA CTCTCGGCGGCAGTCGTAT
256. vanHD GTGGCCGATTATACCGTCATG CGCAGGTCATTCAGGCAAT
257. vanRA-01 CCCTTACTCCCACCGAGTTTT TTCGTCGCCCCATATCTCAT
258. vanRA-02 CCACTCCGGCCTTGTCATT GCTAACCACATTCCCCTTGTTTT
259. vanRB GCCCTGTCGGATGACGAA TTACATAGTCGTCTGCCTCTGCAT
260. vanRC TGCGGGAAAAACTGAACGA CCCCCCATACGGTTTTGATTA
261. vanRC4 AGTGCTTTGGCTTATCTCGAAAA TCCGGCAGCATCACATCTAA
262. vanRD TTATAATGGCAAGGATGCACTAAAGT CGTCTACATCCGGAAGCATGA
263. vanSA CGCGTCATGCTTTCAAAATTC TCCGCAGAAAGCTCAATTTGTT
264. vanSB GCGCGGCAAATGACAAC TTTGCCATTTTATTCGCACTGT
265. vanSC-02 GCCATCAGCGAGTCTGATGA CAGCTGGGATCGTTTTTCCTT
266. vanSE TGGCCGAAGAAGCAGGAA CAATAATACTCGTCAAAGGAGTTCTCA
267. vanTC-01 CACACGCATTTTTTCCCATCTAG CAGCCAACAGATCATCAAAACAA
268. vanTC-02 ACAGTTGCCGCTGGTGAAG CGTGGCTGGTCGATCAAAA
269. vanTE GTGGTGCCAAGGAAGTTGCT CGTAGCCACCGCAAAAAAAT
270. vanTG CGTGTAGCCGTTCCGTTCTT CGGCATTACAGGTATATCTGGAAA
271. vanWB CGGACAAAGATACCCCCTATAAAG AAATAGTAAATTGCTCATCTGGCACAT
272. vanWG ACATTTTCATTTTGGCAGCTTGTAC CCGCCATAAGAGCCTACAATCT
273. vanXA CGCTAAATATGCCACTTGGGATA TCAAAAGCGATTCAGCCAACT
274. vanXB AGGCACAAAATCGAAGATGCTT GGGTATGGCTCATCAATCAACTT
275. vanXD TAAACCGTGTTATGGGAACGAA GCGATAGCCGTCCCATAAGA
276. vanYB GGCTAAAGCGGAAGCAGAAA GATATCCACAGCAAGACCAAGCT
277. vanYD-01 AAGGCGATACCCTGACTGTCA ATTGCCGGACGGAAGCA
278. vanYD-02 CAAACGGAAGAGAGGTCACTTACA CGGACGGTAATAGGGACTGTTC
279. vatB-01 GGAAAAAGCAACTCCATCTCTTGA TCCTGGCATAACAGTAACATTCTGA
280. vatB-02 TTGGGAAAAAGCAACTCCATCT CAATCCACACATCATTTCCAACA
281. vatC-01 CGGAAATTGGGAACGATGTT GCAATAATAGCCCCGTTTCCTA
282. vatC-02 CGATGTTTGGATTGGACGAGAT GCTGCAATAATAGCCCCGTTT
283. vatE-01 GGTGCCATTATCGGAGCAAAT TTGGATTGCCACCGACAAT
284. vatE-02 GACCGTCCTACCAGGCGTAA TTGGATTGCCACCGACAATT
285. vgaA-01 CGAGTATTGTGGAAAGCAGCTAGTT CCCGTACCGTTAGAGCCGATA
286. vgaA-02 GACGGGTATTGTGGAAAGCAA TTTCCTGTACCATTAGATCCGATAATT
287. vgb-01 AGGGAGGGTATCCATGCAGAT ACCAAATGCGCCCGTTT
288. vgbB-01 CAGCCGGATTCTGGTCCTT TACGATCTCCATTCAATTGGGTAAA
289. vgbB-02 ATACGAGCTGCCTAATAAAGGATCTT TGTGAACCACAGGGCATTATCA
290. yceE/mdtG-01 TGGCACAAAATATCTGGCAGTT TTGTGTGGCGATAAGAGCATTAG
291. yceE/mdtG-02 TTATCTGTTTTCTGCTCACCTTCTTTT GCGTGGTGACAAACAGGCTTA
292. yceL/mdtH-01 TCGGGATGGTGGGCAAT CGATAACCGAGCCGATGTAGA
293. yceL/mdtH-02 CGCGTGAAACCTTAAGTGCTT AGACGGCTAAACCCCATATAGCT
294. yceL/mdtH-03 CTGCCGTTAAATGGATGTATGC ACTCCAGCGGGCGATAGG
295. yidY/mdtL-01 GCAGTTGCATATCGCCTTCTC CTTCCCGGCAAACAGCAT
296. yidY/mdtL-02 TGCTGATCGGGATTCTGATTG CAGGCGCGACGAACATAAT
