# Supplementary material for: Impacts of cellulase and xylanase addition on antibiotic resistance and microbial community during dairy manure composting
Source: PLoS One. 2025 Aug 29;20(8):e0328055. doi: 10.1371/journal.pone.0328055 (PMC12396641; doi:10.1371/journal.pone.0328055)
Supplement: Attachment — Submitted filename: PONE-D-24-53884 reviewed.pdf [file pone.0328055.s003.pdf]

Manuscript Number: PONE-D-24-53884

Title: Impacts of cellulase and xylanase addition on antibiotic resistance and microbial community during dairy manure composting.

Overview and general recommendation:

This subject is important because it evaluated the effects of cellulase and xylanase on the physicochemical properties, microbial community, and antibiotic resistome.

I found the manuscript well written, well described and the references were new.

I listed **major** and **minor points** below for editing.

Comments to Authors:

Major points

1- In results: you wrote many sentences as discussion!!! The rule of manuscripts' writing is to avoid using any discussing points in the results chapter, so you should transfer those sentences to discussion part.

2- You should add conclusion part at the end of discussion chapter.

Minor points

1- In this manuscript; **you wrote (5 times Our)!!!** The rule of manuscript writing is to avoid using (Our). So you should delete (Our) and use academic scientific words such as (This study or The current study or The present study).

Kind regards
